# Colonic Perforation: A Case Report of a Rare Delayed Complication Due to Accidental Swallowing of Denture

**DOI:** 10.7759/cureus.26461

**Published:** 2022-06-30

**Authors:** Rameshwar Lal, Dheerain Gupta, Ramkaran Chaudhary, Suresh Rulaniya, Indra s Choudhary

**Affiliations:** 1 General Surgery, Dr. Sampurnanand Medical College, Jodhpur, IND; 2 General Surgery, All India Institute of Medical Sciences, Jodhpur, IND; 3 Urology & Renal Transplant, Srirama Chandra Bhanja Medical College & Hospital, Cuttack, IND

**Keywords:** explorative laparotomy, complication, foreign body, denture, intestinal perforation

## Abstract

Removable partial dentures are the most common object found in elderly patients presenting with a history of foreign body ingestion. These patients will usually present within a week with complications if the foreign body gets impacted in the gastrointestinal tract. In this case report, we present a rare delayed complication in the form of colonic perforation that presented three years after swallowing a denture, with the emphasis on how to suspect and manage these patients.

## Introduction

Artificial teeth in the form of crowns, dental implants, or removable dentures are extremely common. Removable partial dentures account for 4% to 18 % of foreign body ingestion and are the most commonly found object found in elderly patients [1]. Impaction of these ingested dentures can occur anywhere along the alimentary canal but is most commonly found in the esophagus followed by the large bowel [2]. Complications such as bleeding, necrosis, perforation, or penetration into the adjacent organ forming fistula although rare, can occur. Here, we present a rare delayed complication in the form of colonic perforation three years after accidentally ingesting a denture.

## Case presentation

A 71-year-old female presented to our emergency department with generalized pain in the abdomen and distension for three days. The symptoms worsened over time. The history revealed that the patient accidentally swallowed her denture three years before presentation. An upper gastrointestinal (GI) endoscopy was not performed at the initial time. According to the patient’s attendant, an X-ray of the abdomen was done at that time which was reported to be normal and she remained asymptomatic thereafter with no signs and symptoms of small bowel obstruction. She had a history of total abdominal hysterectomy for symptomatic fibroid 15 years ago. On examination, the patient was well built and oriented. She was febrile and had a blood pressure of 100/60 mmHg, pulse rate of 118/min, and respiration rate (RR) of 22/min. Her abdomen was distended and on palpation, generalized tenderness and guarding were elicited. The resuscitation of the patient was started and investigations were sent as per our institute’s protocol.

**Figure 1:**
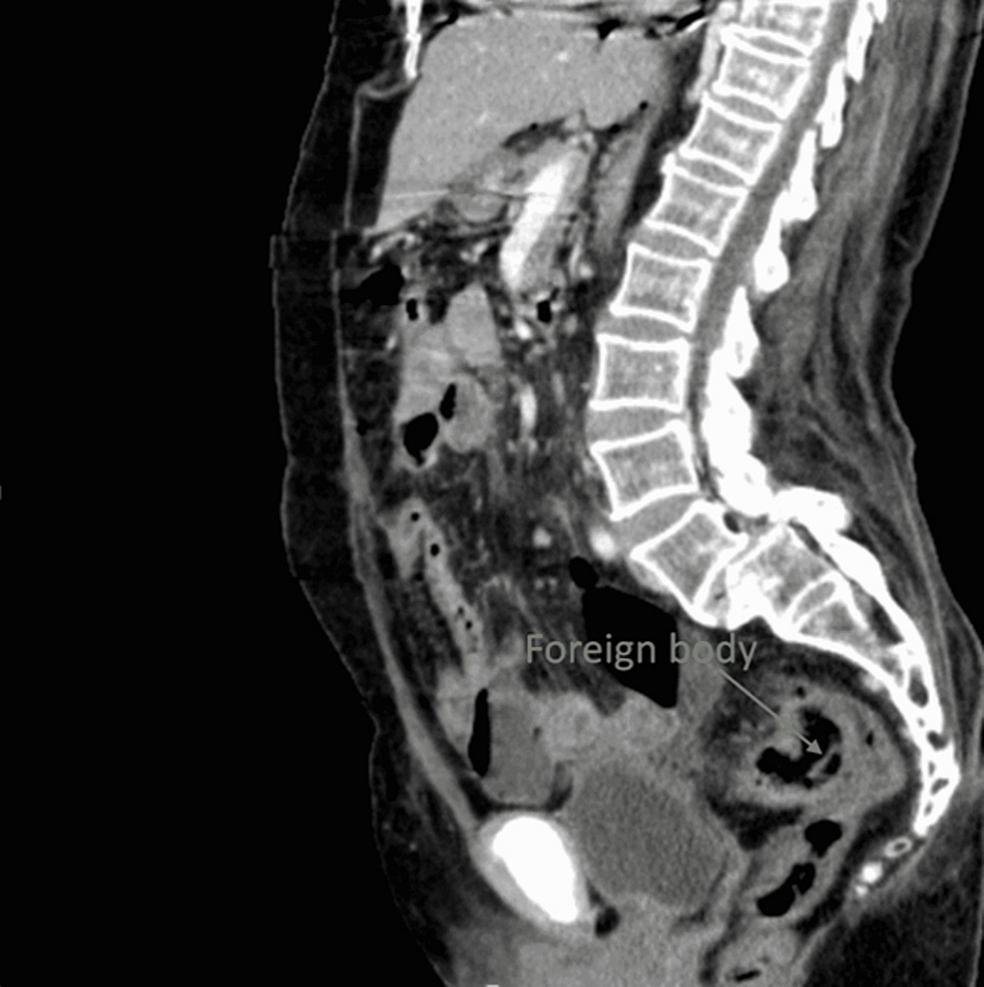
CECT of the abdomen and pelvis (sagittal view) showing visible partial denture at the rectosigmoid junction CECT: High-dose contrast-enhanced computed tomography

The biochemical investigation showed WBCs of 10730 per microliter, hemoglobin 12.8 gm/dl, platelets 222 x109 /L, serum creatinine 1.34 mg/dl, and serum amylase 18 units/liter. A high-dose contrast-enhanced computed tomography (CECT) of the abdomen and pelvis revealed pneumoperitoneum and a foreign body at the rectosigmoid junction with mild bilateral pleural effusion (Figure 1) but was unable to identify the site of perforation.

**Figure 2:**
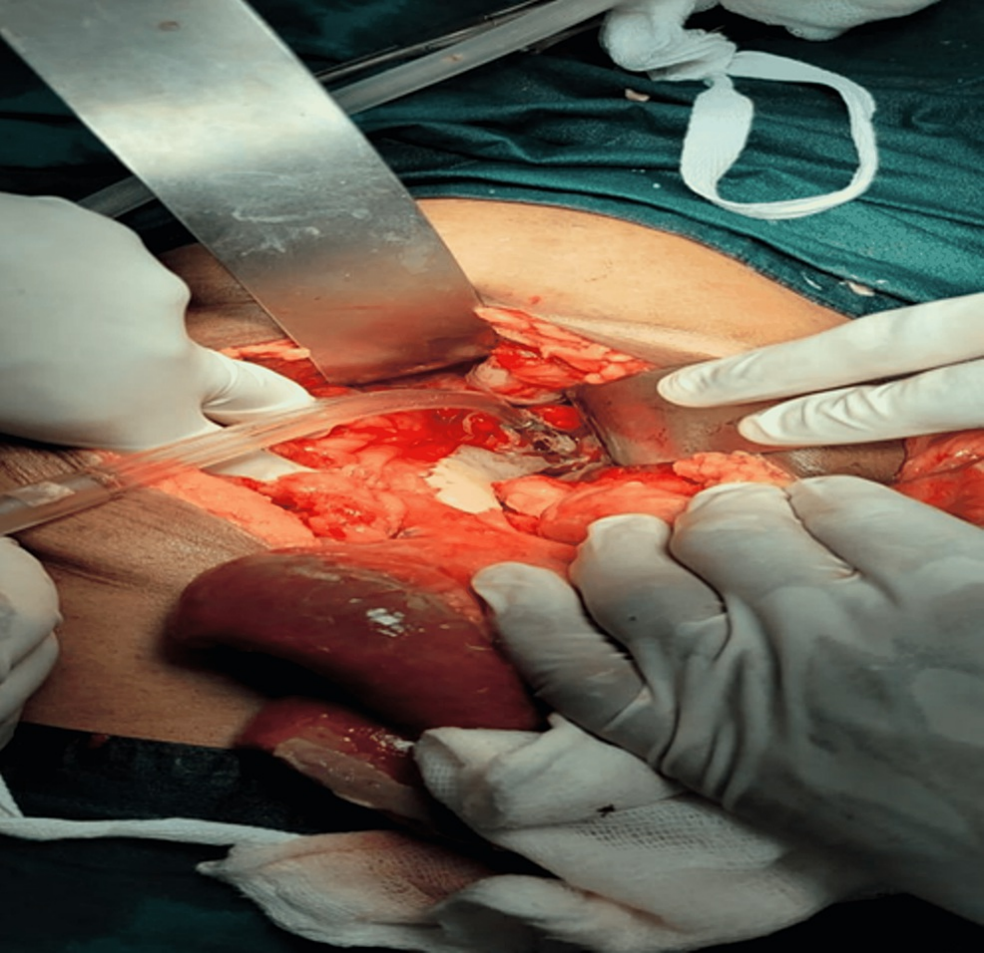
Intraoperative perforation site at the distal sigmoid colon

**Figure 3:**
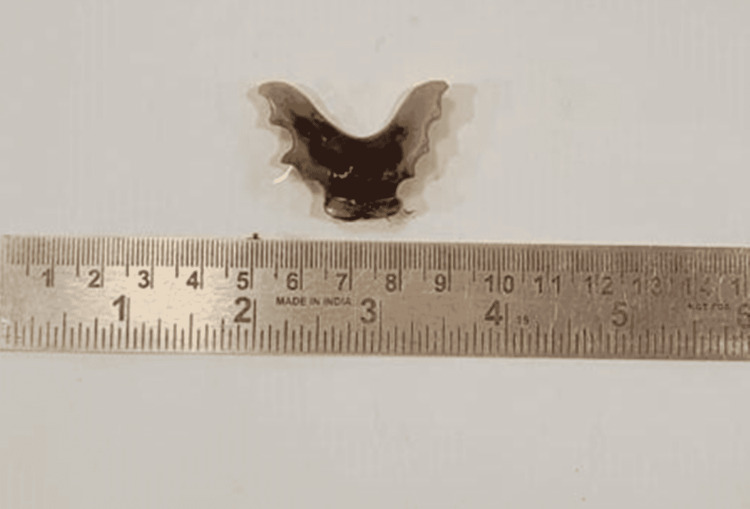
Postoperative specimen of the denture

A working diagnosis of small bowel perforation peritonitis was made. An emergency diagnostic laparoscopic surgery was performed which converted to a laparotomy. Intraoperative findings showed pneumoperitoneum with a 1x1 cm perforation at the antimesenteric border of the distal sigmoid colon (Figure 2) with protrusion of a partial denture that was removed as a specimen (Figure 3).

After thorough lavage with saline, primary repair of the perforation with a defunctioning loop ileostomy was done. Postoperatively the patient was shifted to the ward but on postoperative day two, she developed atrial fibrillation. So she was further managed in the ICU. The patient improved surgically after that with no further complications. Stoma became functional on postoperative day three. She was discharged on postoperative day 14 with a well-functioning stoma in situ. Stoma closure was done at six weeks. On further follow up she was doing well.

## Discussion

The base of removable partial dentures is made of a radiolucent plastic material known as polymethylmethacrylate, whereas the tooth is made of porcelain. The radiopaque area of a denture is the metal pin that holds it together. So it could be easily overlooked in the plain radiograph and requires high suspicion for the diagnosis. Though the plain X-ray is recommended as the first investigation, a multiplanar CT is the investigation of choice to mark the exact location of the ingested denture [3].

The passage through the alimentary canal depends on the diameter as well as the length of the ingested foreign body [1]. A recent review by Kent et al. concluded that all shapes (hooked or unhooked) of dentures carry equal risk of complications like perforation once impacted [4]. The esophagus is the most common site of foreign body impaction. Once in the stomach, 80% to 90% of the time it will pass out spontaneously [5]. Out of those impacted in the upper GI tract, 70% to 75 % can be taken out via rigid endoscopy, and only a few will require operative intervention [6], but the same is not true with impaction in the small or large bowel which requires early operative intervention to avoid serious complications.

Most times a foreign body passes through the alimentary canal spontaneously in three to five days without any complications and if not, it will present with complication mostly with perforation within a week [7]. But in our case, the patient remained asymptomatic for almost three years. In the literature reviewed, none have reported latency of years before presentation. It could be subjected to the recall bias but it was confirmed multiple times by three different doctors and there was no recent history of any loss or ingestion of dentures.

## Conclusions

Any elderly patient who presents with acute abdomen and a history of ingestion of denture material even years before should be suspected of foreign body impaction-related complications. Patients should be evaluated with a contrast-enhanced CT scan to locate the foreign body and early surgical intervention should be considered for the removal of the foreign body impacted in the lower GI tract.
